# Resistance Mechanisms to CAR T-Cell Therapy and Overcoming Strategy in B-Cell Hematologic Malignancies

**DOI:** 10.3390/ijms20205010

**Published:** 2019-10-10

**Authors:** Moo-Kon Song, Byeong-Bae Park, Ji-Eun Uhm

**Affiliations:** 1 Department of Hemato-Oncology, Hanyang University Hanmaeum Changwon Hospital, Changwon 51497, Korea; song9676@hanmail.net; 2 Division of Hematology-Oncology, Department of Internal Medicine, Hanyang University College of Medicine, Hanyang University Seoul Hospital, Seoul 04763, Korea; jieunuhm@hanyang.ac.kr

**Keywords:** CAR T-cell, drug resistance, B cell hematologic malignancies

## Abstract

Chimeric antigen receptor (CAR) T-cell therapy has shown promising clinical impact against hematologic malignancies. CD19 is a marker on the surface of normal B cells as well as most B-cell malignancies, and thus has a role as an effective target for CAR T-cell therapy. In numerous clinical data, successes with cell therapy have provided anticancer therapy as a potential therapeutic option for patients who are resistant to standard chemotherapies. However, recent growing evidence showed the limitations of the treatment such as antigen-positive relapse due to poor CAR T-cell persistence and antigen-negative relapses associated with CAR-driven mutations, alternative splicing, epitope masking, low antigen density, and lineage switching. The understanding of the resistance mechanisms to the cell therapy has developed novel potential treatment strategies, including dual-targeting therapy (dual and tandem CAR), and armored and universal CAR T-cell therapies. In this review, we provide an overview of resistance mechanisms to CD19 CAR T-cell therapy in B-cell malignancies and also review therapeutic strategies to overcome these resistances.

## 1. Introduction

Chimeric antigen receptor (CAR) is a synthetic tumor-specific receptor that can bind to target cell surface antigens via a single-chain variable fragment (scFv) recognition domain, hinge regions, a transmembrane domain, and an intracellular signaling domain transmitting activation signals [1,2,3]. Several previous studies investigated CAR T-cell therapy for B-cell hematologic malignancies [4,5,6,7,8,9,10,11,12,13,14,15,16,17,18,19]. The results demonstrated favorable results by targeting CD19, CD20, or CD30, and the most promising outcomes have been achieved in CD19-specific CAR T-cells for B-cell acute lymphoblastic leukemia (B-ALL) with a high complete remission (CR) rate of 70–94% [10,11,12,13,14,15]. Targeting CD19 CAR positive tumor cells represents a paradigm change in the therapeutic strategy of B-cell malignancies, resulting in a strong impetus for the expanded application of the cell therapy in T-cell malignancies and solid tumors.

CD19 is a B-cell specific cell surface marker playing a crucial role in the cell development in normal tissues. It is expressed on the cell surface starting from the early stages of B-cell lineage and lost during maturation to plasma cells. Acting as a B-cell co-receptor, CD19 not only supports early B-cell development but also mediates the maturation of peripheral blood B cells [20,21]. Thus, it is a prospective antigen for CAR T-cell therapy. Recently, some clinical data of the cell therapy of relapsed or refractory CD19-positive B-cell malignancies demonstrated excellent long-term remission, and patients receiving the treatment were potentially cured [10,11,12,13,14,15,16,17,18,19]. However, 30–50% of patients who achieve complete remission (CR) after the cell therapy will experience relapse of disease, mostly within 1 year of treatment [11,14]. Moreover, about 10–20% of patients do not achieve CR after the therapy [11,12,13,14]. 

Active CAR T-cell-mediated immune surveillance plays an important role in durable remission after the cell therapy [10]. Loss of the CAR T-cell persistence may be an important determinant of antigen-positive relapse. Meanwhile, immune pressure by CAR T-cells leads to the modulation of antigen expression by cancers via the loss of a detectable antigen or diminished antigen density to the level below a threshold required for the cell activity. Recently, the proliferation of CD19-negative tumor cells has been reported in both pediatric and adult responders exposed to the CAR T-cell therapy in B-ALL [10,11,12,13,14,15]. In this review, we will review the various mechanisms of resistance to the therapy in B-cell hematologic malignancies. 

## 2. The Role of CD19 CAR T-Cell Therapy in B-Cell Malignancies

Recent clinical data demonstrated about 70–90% of pediatric B-ALL patients achieved had a similar overall response rate and impressive results following the CAR T-cell therapy that was reported in adults (Table 1) [10,11,12,13,14,15]. However, outgrowth of the antigen escape may decrease the durability of response in patients undergoing the treatment despite the durable persistence of CAR T-cells. In a recent phase 1 trial reported by the University of Pennsylvania and Children’s Hospital of Pennsylvania (CHOP), 3 of 27 responders (11%) relapsed with B-ALL without detectable CD19 [10]. In phase II ELIANA trial of Novartis’s tisagenlecleucel, which is a synthetic bio-immune product of anti-CD19 CAR T-cells, at least 61 of 75 pediatric and young adult B-ALL patients (81%) achieved CR and 15 of the responders (24.6%) went on to develop the antigen-negative or partially negative relapse [11]. In addition, Lee et al. showed that CR was 66.7%, and 14.3% developed antigen-negative relapse [12]. Clinical data reported by Seattle Children’s Research Institute showed that 2 of 7 pediatric and adult patients (18%) who achieved CR, relapsed with lineage switch due to the antigen loss [13]. Similarly, the results from Memorial Sloan Kettering Cancer Center (MSKCC) demonstrated that 4 of 44 adult B-ALL patients (9%) showed a disease relapse with the antigen loss [14].

Recent phase II studies reported the response data involving relapsed or refractory B-cell non-Hodgkin’s lymphoma (B-NHL). The phase II clinical study (ZUMA-1) of axicabtagene ciloleucel (autologous anti-CAR T-cell therapy) for relapsed or refractory B-NHLs reported that 3 of 11 patients (27.2%) with disease progression tested negative for CD19 at the time of disease progression (Table 1) [16]. Jacobson et al. evaluated the clinical impact of tisagenlecleucel in patients with relapsed diffuse large B-cell lymphoma. Samples obtained from four relapsed patients after the cell therapy were analyzed for the CD19 loss, and antigen-negative disease was detected in one case [18]. Similar data were reported by Oak et al. [19]. 

The abovementioned data presented in Table 1 showed that the cell therapy had superior efficacies in B-ALL compared with in B-NHL. Moreover, the excellent response rate of the cell therapy was very impressive when compared with previous conventional treatments used for patients diagnosed with relapsed or refractory B-cell malignancies. Thus, it showed a promising role as an optimal treatment modality for patients with relapsed or refractory B-cell malignancies. However, the disease relapse following the cell therapy can occur in a few patients after infusion. The results showed that one patient showed antigen-positive relapse, while relapse in another case was associated with antigen loss. To overcome the relapse after the treatment, it is necessary to understand the resistance mechanisms of cancer cells after infusion. 

## 3. Cancer Cell Killing Mechanisms by CAR T-Cells

Cancer cell lysis by CAR T-cells is classified into three killing mechanisms: Fas/Fas ligand (FasL) pathway-associated tumor cell lysis, a cytokine-induced killing mechanism, and tumor cell lysis by granzyme and perforin. 

Fas/FasL pathway was known to be associated with both beneficial and harmful effects of effector T cells [20,21]. The calcium-independent Fas/FasL pathway is an important mechanism for T cells to kill tumor cells [22,23,24]. By trimerization of the Fas receptor by FasL, caspase 8 is activated and thus downstream pro-caspase 3 could be mature caspase 3, which would mediate tumor cell lysis through downstream pathways [25,26,27]. Recent data showed that activation of CD30 and CD90 targeting CAR T-cells could induce tumor lysis against the antigen-negative portion in an antigen-independent, cell-to-cell contact-mediated manner [28]. The data suggested that CD30 CAR T-cells might be useful to kill cancer cells. Additionally, Fas/FasL interaction between CAR T-cells and cancer cells is able to reduce tumor escape due to heterogeneous antigen expression or to enhance the antitumor capacity. 

In addition, cytokine production by activated CAR T-cells enhances antitumoral activity. Cytokine secretion by the cells has a crucial role in inducing tumor lysis through stromal sensitization and macrophage polarization CAR T-cell killing. Especially, IL-12 was showed to activate antitumor immune reactions [29,30]. Moreover, the mechanism is associated with improvement of T-cell cytolytic activity [31], the recruitment and activation of innate immune cells, and the reprogramming of stroma-associated immune suppressor cells. 

Furthermore, the perforin and granzyme pathway is another important mechanism for CAR T-cell induced tumor cell lysis. Perforin mediates pore formation on the cancer cell membrane to promote the access of pro-apoptotic granzyme. Once in the cytoplasm of the target cell, granzyme can mediate apoptotic cell death by cleaving the key substrates [32,33]. 

## 4. Antigen-Positive Relapse after CD19 CAR T-Cell Therapy

Poor CAR T-cell persistence or transient B-cell aplasia indicates an inconsistent effect of immunotherapy, which is often associated with an early relapse of leukemic or lymphoma cells within a few months after induction of successful remission. CAR T-cell persistence is determined by inherent T-cell quality, initial T-cell phenotype, and co-stimulatory domain in each CAR construct [34,35,36].

Kotani et al. showed that CAR T-cells extracted from aged mice exhibit cytotoxicity but shorter persistence and few memory-like phenotypes [37]. The data suggested that the differences in clinical outcomes between younger and elder patients might be attributed to an age-dependent phenotype of the cell that is reflected by the unique pattern of gene expression, secretory profile, and/or transcription factor balance. 

Both CD4+ and CD8+ cytotoxic T lymphocytes effectively kill cancer cells using the cytolytic perforin and granzyme [38,39,40]. Thus, CAR T-cells rapidly destroy and eliminate target cells via degranulation of the proteins. Compared with CD8+ cells, CD4+ CAR T-cells contain lower levels of intracellular perforin and granzymes [41,42]. Therefore, target cell lysis by CD4+ CAR T-cells is delayed or requires a higher number of effector cells for effective lysis compared with CD8+ T-cells. Based on this perspective, the initial T-cell phenotype such as the percentage of CD4+ and CD8+ CAR T-cells determines cell persistence. 

The second-generation CAR containing a TCR stimulatory domain such as glycoprotein CD3-ζ chain (CD3-ζ) and a single co-stimulatory domain provides signals for activation of T-cells [43]. As mentioned above, CAR T-cell persistence relies on the type of co-stimulatory domain. CD28 and 4-1BB have been known as the popularly tested co-stimulatory domains. The 4-1BB co-stimulatory domain-containing CAR T-cells were associated with longer persistence than the CD28 co-stimulatory domain [11]. Moreover, 4-1BB domain-containing CAR T-cells were showed to have a lower tendency for T-cell exhaustion than those containing CD28 [44]. 

## 5. Antigen-Negative Relapse 

**Figure 1:**
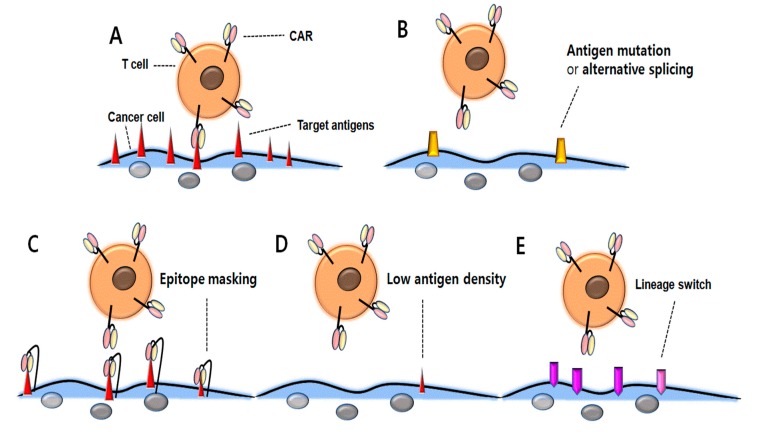
Resistance mechanisms associated with antigen loss following CD19 CAR T-cell therapy. (**A**) CAR T-cells bind with tumor-associated antigens and represent antitumor activity. (**B**) Due to CD19 CAR mutations or alternative splicing, CAR T-cells cannot bind the antigens and therefore, cancer cells may be resistant to therapy. (**C**) Due to CD19 CAR binding to CD19 antigen and subsequent masking of the CD19 epitope, CAR T-cells cannot attack the cancer cells. (**D**) Down-regulated antigen density prevents cancer cells against CD19 CAR T-cell therapy. (**E**) The surface antigen is changed from lymphoid to myeloid lineage, which prevents CD19 CAR T-cells from binding to the cancer cells.

Recent data of CD19 CAR-T cells demonstrated several possible mechanisms of antigen escape. Despite highly promising results of the cell therapy in B-cell malignancies, antigen escape relapse has emerged as a serious challenge for the maintenance of long-term remission. Continuous therapeutic stress in highly-specific CAR T-cells might lead to the development of several possible mechanisms of antigen escape, such as alternative forms of the receptor via antigen mutations or alternative splicing, decreased antigen density, epitope masking, and lineage switching (Figure 1). Here we will review the potential mechanisms for relapse associated with antigen loss and novel therapeutic options to combat this challenge.

## 6. CAR-Driven Acquired CD19 Mutations 

Frameshift mutations strongly induce genetic inactivation particularly when premature stop codons are closely inserted to the 5′ ends of open frames [45]. The mutations could lead to antigen loss through nonsense-mediated mRNA decay or truncated polypeptide chains. 

Sotillo et al. investigated whole-exome sequencing, RNA sequencing and copy-number alteration analysis [46]. The findings showed two dependent frameshift mutations in exons 2 and 4 in the sample CHOP101R. As another sample post-CD19 CAR T-cell therapy, the CHOP133R sample showed a hemizygous loss of chromosome 16 and a frameshift mutation in exon 2 leading to nonsense-mediated decay. 

In other data by Orlando et al., de novo frameshift mutations mapping exon 2 to 5 were found in the samples of 12 patients relapsing after the cell therapy and copy number analysis confirmed heterozygous antigen loss in 8 of 9 patients [47]. Ultimately, the data revealed that the antigen loss due to the frameshift mutation altered the regulation of transcript processing.

## 7. Alternative Splicing of CD19

Alternative splicing is a post-transcriptional mechanism leading to the production of alternative mRNA transcripts that encode structurally and functionally different protein isoforms [48]. Therefore, it increases protein diversity and plays an important role in human development. Meanwhile, cellular plasticity via alternative splicing encourages cancer cells to produce protein isoforms associated with tumor cell proliferation, invasion, and spreading. Alternative splicing of CD19 may affect the protein function associated with cell signaling and B-cell function at different stages of B-cell maturation. 

In data by Sotillo et al., molecular events associated with obvious CD19 antigen loss in patients experienced disease relapse after the CAR T-cell therapy [46]. The results showed that two patients had increased levels of the isoform lacking exon 2 (Δex2). The Δex2 plays a crucial part in the alteration of the cognate epitope necessary for CAR recognition. Therefore, the isoform reflects resistance to CAR T-cell targeting and/or death of leukemic cells. 

Similarly, the splicing variant with loss of exon 16 in the extracellular domain of HER2 showed therapeutic resistance to trastuzumab [49,50]. Moreover, the aberrant splicing of BRAF V600E was associated with BRAF inhibitor, and vemurafenib treatment [51,52]. In addition, the skipping of exon 5 or 6 (Δex5, Δex6) with a loss of transmembrane and cytosolic domains, was associated with the immune evasion of the tumor cells under therapy [46]. 

It will be important to analyze the causal factors of CD19 exon splicing and its relationship with the therapeutic outcome. The causal factors of the splicing include genetic mutations and single nucleotide polymorphisms that affect exon skipping and change the expression level of splicing factors. Several splicing factors, including SF3B1, U2AF1, SRSF2, and SRSF3, have been identified in chronic lymphocytic leukemia, myelodysplastic syndrome, lymphoma, and ALL [48,53,54,55]. 

Sotillo et al. evaluated the relationship between splicing factor and exon 2 alternative splicing using four small interfering RNA pools in B-lymphoid P493-6 cell lines [46]. The results of the quantitative RT-PCR assay showed that only SRSF3 knockdown, among several splicing factors, affects Δex2. Notably, SRSF3 knockdown resulted in high expression of Δex2 protein isoform in both P493-6 and NALM-6 B-ALL cells by immunoblotting measurement. Moreover, relapsed leukemia cells expressed lower amounts of SRSF3. However, inadequate SRSF3 in relapsed leukemia cells was at least partly responsible for the expression of the Δex2 isoform. 

## 8. Masking of CD19 Epitope 

Recently, Ruella et al. demonstrated the relapse mechanism of a patient with B-ALL after CAR T-cell therapy [56]. The patient showed bone marrow involvements of aberrant antigen-negative leukemic cells and circulating CAR-transduced B-cells with leukemia. Although CD19 protein was not detected in flow cytometry, the transcripts of the proteins were identified in the baseline apheresis and at relapse. Therefore, the authors assumed that the lack of CD19 detection was due to CAR binding to the antigen and the subsequent epitope masking. The authors confirmed that the CAR gene was unintentionally introduced into a single leukemic B-cell during the CAR T-cell manufacturing process and the product bound in cis to the CD19 epitope on the surface of leukemic cells. 

## 9. Low Antigen Density 

Unlike signaling mediated via the T-cell receptor (TCR) in response to very low antigen density, the engineered CAR T-cells need a higher density of the antigen to fully enhance effector functions [57]. Therefore, tumor variants with low antigen density may represent a resistance mechanism in the cell therapy. Recently, Walker et al. investigated the utility of CAR T-cell therapy by targeting anaplastic lymphoma kinase (ALK) in pediatric solid tumors [58]. The activation of CAR T-cells was insufficient because of the low target density of ALK on the cell lines. In addition, antitumor efficacy of the ALK CAR was limited by the diminished target antigen and CAR density in the xenograft model.

CD30, as an alternative target of various lymphoma cells, was investigated in lymphoma therapy. In addition, lymphocyte and hematopoietic stem and progenitor cells (HSPCs) express lower levels of CD30 than cancer cells. Hombach et al. showed that anti-CD30 CAR T-cell therapy did not attack normal HSPCs while eliminating CD30-positive lymphoma cells [59]. The data demonstrated that HSPCs with lower CD30 levels may be protected against the cell therapy due to the lower antigen density. 

Moreover, Watanabe et al. determined the threshold of target antigen density to induce the response using new developed CD20 CAR T-cells containing CD28 intracellular domain and a CD20-transduced cell model [60]. The data showed that antigen density for cytokine production of CAR T-cells is ten-fold higher than the threshold of antigen density for lytic activity. Moreover, they found that rituximab and ofatumumab were not effective against CD20-downregulated leukemia and lymphoma cancer cells, although CD20 CAR T-cell therapy was effective, which suggested that the very low CD antigen density affected the response rate of monoclonal antibody therapy effectively. 

CD22 is a sialic acid-binding immunoglobulin-like lectin which is expressed in numerous B-cell malignancies. It is also expressed in most B-ALL cases and is usually retained following CD19 loss. In a recent phase I study of CD22 CAR in 22 patients, including 17 who were previously treated with CD19 CAR T-cell therapy [61], CR was found in 73% (11/15) of patients including 8 of 10 patients who previously received CD19 CAR T-cell therapy. Interestingly, seven of eight patients who experienced disease relapse after CD22 CAR T-cell therapy had decreased the antigen density or loss. These data suggested lack of association between treatment response and previous therapies such as chemotherapy or CD19-based immunotherapy. However, it showed that a decreased CD22 site density than the total surface loss was more associated with immune escape of leukemia from the CAR T-cell therapy. 

Ramakrishna et al. also evaluated the implications of antigen density for CD22 CAR T-cell therapy and proposed mechanisms to overcome antigen escape [62]. The data demonstrated that low CD22 density had similar negative impacts in vitro and in vivo on CD22 CAR T-cell function and impaired in vivo CAR T-cell persistence in ALL cell lines with variable CD22 expression. 

## 10. Lineage Switching 

The switching in cell lineage is another mechanism associated with antigen escape. It entails a complete switch of leukemic cells from B-cell lineage to myeloid lineage after CAR T-cell therapy [45,63]. Usually, the lineage switch occurs in B-ALL or acute leukemia with mixed phenotype carrying mixed-lineage leukemia (MLL) gene rearrangement on chromosome 11q23 [13,64,65,66].

Evans et al. first reported that a patient with relapsed and refractory chronic lymphocytic leukemia with Richter transformation received CD19 CAR T-cell therapy, and later relapsed into plasmablastic lymphoma without CD19 expression [67]. Similarly, Gardner et al. found that two of seven B-ALL patients with MLL after the cell therapy developed acute myeloid leukemia at the time of relapse [13]. Jacoby et al. also demonstrated immune pressure against CD19 by CAR T-cells at the cellular level resulting in lineage switch of leukemia due to reprogramming induced by ablation of the B-cell transcription factors resulting in complete arrest of B-cell developmental pathways [66].

## 11. Treatment Strategy to Prevent Antigen-Positive Relapse

As mentioned above, because of the longer persistence of 4-1BB domain-containing CAR T-cells, the therapy may lead to a reduced antigen-positive relapse. A recent study demonstrated that the CD28 co-stimulatory domain-containing CAR T-cells increase gene expressions related to T-cell exhaustion, meanwhile the 4-1BB co-stimulatory domain carrying the same antigen specificity attenuated this exhausted phenotype [30]. Several data suggest that CAR T-cells containing the CD28 domain persist up to 3 months, while those containing the 4-1BB domain persist for up to 5 years [4,12]. Moreover, other data showed that pediatric patients with B-ALL treated with CD19 CAR T-cells incorporating a 4-1BB co-stimulatory domain showed higher rates of minimal residual disease testing negative for CR than those with CD28 domain [68]. 

Recently, the administration of artificial antigen-presenting cells (AAPCs) led to an off-the-shelf approach for effective adoptive T-cell immunotherapy by increasing the expansion and durability of infused CAR T-cells. AAPC has potential advantages to optimize and precisely control the delivery of signals required for CAR-T cell activation and expansion, including signals regulating cell adhesion, co-stimulation, cytokine secretion, and interactions between major histocompatibility complex (MHC)-peptide complexes and the T-cell receptor (TCR) [69]. Moreover, central memory T-cells and stem cell-like memory T-cells induced sustained proliferation and persistence of CAR T-cells after the cell therapy by ensuring essential preconditions for treatment efficacy [70,71]. Thus, procedures inducing the transformation of CAR T-cells to central memory T-cells and stem cell-like memory T-cells could be an alternative for prevention of antigen-positive relapse by enhancing a durable response and persistence of the cells. 

Lymphodepletion also enhances the cell response through the eradication of regulatory T-cells, elimination of other immune cells that compete for homeostatic cytokines and enhancing antigen-presenting cell activation [64,72]. The Fred Hutchinson Cancer Research Center (FHCRC) group of adults with B-ALL and B-NHL showed that transgene rejection may be prevented via adequate lymphodepletion [73,74]. The results showed lymphodepletion via preconditioning therapy containing fludarabine (Flu) combined with cyclophosphamide (Cy) increased CAR T-cell levels and may be associated with improved clinical outcomes. Recently, a small molecule inhibitor, ibrutinib also improved the cell engraftment, tumor clearance, and survival in preclinical and clinical studies of hematologic malignancies [75,76].

In addition, the co-inhibitory pathway-related T-cell exhaustion was thought to contribute to the poor persistence of CAR T-cells [77]. Recent studies showed that the T-cell exhaustion-related pathway was up-regulated in non-responders with B cell hematologic malignancies after the cell therapy [11,78]. In some data, the expression of T cell co-inhibitory receptors including PD-1, Tim-3, and LAG-3 was up-regulated on CAR-T cells resulting in possible inhibitory effects [79]. Another study also showed that PD-1 or LAG-3-deficiency could improve antitumor efficacy of the cell therapy [80]. 

The PD-L1/PD-1 pathway is another CAR T-cell exhaustion-related pathway, which directly down-regulated the signaling of the CD28 co-stimulatory domain and induced the cell dysfunction [79,81]. Therefore, the combination therapy of immune checkpoint inhibitors and CAR T-cells represents another possible strategy for CAR T-cell expansion and persistence. 

Antigen-positive relapse also presents the possibility of re-infusion of CAR T-cells to enhance the persistence. However, clinical data supporting re-treatment with CAR T-cells only showed a limited efficacy for the treatment of antigen-positive relapse [82]. 

Injection of attenuated viruses, such as vaccinia or viral antigens to host cells, stimulates the native TCR and promotes T-cell activation. However, several clinical data have not shown any clear evidence. 

## 12. Overcoming Resistance to CAR T-Cell Therapy 

Numerous studies demonstrated the efficacy of CD19 CAR T-cell therapy in hematologic malignancies. However, antigen escape after the cell therapy against cancer cells is an emerging issue. Thus, various approaches against antigen escape by cancer cells were also introduced in several studies. 

Based on an understanding of various resistance mechanisms involved in CD19 antigen loss, CARs designed to target alternative epitopes on CD19 may not be effective, as many cases of antigen loss are associated with a loss of CD19 surface antigen expression. Therefore, to overcome the antigen loss as a resistance mechanism in immunotherapy, CAR constructs that target multiple antigens including CD19 are designed to resolve the inherent tumor heterogeneity and thus decrease the relapse rate of cancer. 

## 13. Dual and Tandem CARs against Antigen Loss

One strategy to diminish the relapse rate from antigen loss following CAR T-cell therapy is the development of dual CARs, which target two different cancer antigens (Table 2) [83,84]. The dual CARs can be classified into “bi-cistronic CAR” type expressing two CARs, such as CD19 and CD22, simultaneously, and “mono-CAR” type expressing two CARs separately. 

A recent study investigated the clinical efficacy of bispecific CAR including CD19 and CD22 in 13 relapsed or refractory B-ALL pediatric patients [85]. The overall response rate was 90% and 100% minimal residual disease (MRD)-negative complete response (CR) was achieved in six patients who received higher CAR T-cell doses. No relapse was detected with loss of CD19 or CD22 antigen to date. Schultz et al. reported four pediatric relapse or refractory B-ALL cases testing positive and achieving CR on day 28 after the bispecific CAR T-cell therapy with three cases of MRD-negative CR [86]. Other data showed that dual CAR CD19 and CD123 CAR overcame both antigen escape and lineage switch, because CD123 is expressed in both myeloid and B-cell lymphoid malignant cells [83].

Another approach is the clinical application of tandem CAR as a single CAR molecule incorporating two different binding scFv domains [76,87,88]. The single-molecule recognizes multiple antigens in order to enhance the immune synapse. In designing tandem CAR, the position of the target antigen determines the orientation of each binder relative to the membrane. Recent data of tandem CAR targeting also showed a possible clinical benefit in malignancy [89]. 

Dual or tandem CARs to the cognate antigen are enough to drive complete T-cell activation and exhibit antitumor efficacy compared with a pooled combination of CAR T-cells [83,88,90]. The enhanced T-cell function may be observed when both targets are recognized. However, the CARs are associated with an increased rate of toxicity. 

## 14. Armored CAR T-Cells to Improve Antitumor Immunity

To improve antitumor and immune activity, armored CAR T-cells were designed to co-express other molecules, such as cytokines and co-stimulatory molecules (Table 2) [91]. Armored CAR T-cells release immunostimulatory cytokines including IL-12 and express co-stimulatory molecules including CD40L and 4-1BBL. IL-12 released by dendritic cells, macrophages, and neutrophils enhances the cytotoxic activity of CD8+ T-cells and NK-cells, and stimulates the Th1 helper T-cell response. Moreover, it overcomes immune suppression by regulatory T-cells and myeloid-derived suppressor cells [92].

CD40L expressed in armored CAR T-cells activates dendritic cells as well as modulates the tumor phenotype, resulting in enhanced immune surveillance of cancer cells [93,94]. Moreover, the expression of other co-stimulatory molecules such as 4-1BBL or CD80 on the cells also has immunostimulatory effects [95]. This effect may facilitate epitope spreading and reduce the risk of an outgrowth of antigen escape variants.

T-cells redirected for universal cytokine killing (TRUCKs) are CAR-redirected T-cells used as vesicles to produce and release a transgenic product that accumulates in the targeted tissue. This approach facilitates the controlled and site-directed delivery of effector molecules within the tumor tissue [96]. Simultaneously, the TRUCK approach targets tumor-associated antigens in an MHC-unrestricted manner [97] TRUCKs activate the antitumor response of cytotoxic T-cells, the recruitment and activation of innate immune cells and reprogramming of stroma-associated immune suppressor cells [31,97,98,99]. 

## 15. Universal CAR T-Cell Approaches

Currently, patient-derived autologous T-cells are defective in function. Therefore, a new strategy using a universal off-the-shelf ready-to-use therapeutic CAR T-cells is needed to overcome antigen loss or antigen mutation. 

Autologous CAR T-cell therapy is limited by antigen specificity and scalability since each CAR T-cell system targets only one or two antigens [83]. Versatile CARs, such as universal CARs, have the potential possibility to target multiple tumor-associated antigens without re-editing and T-cell production.

Universal CAR T-cell is a tumor antigen-specific T-cell derived from allogeneic healthy donors, which effectively abolish graft-versus-host disease (GVHD) by genetically disrupting TCR gene and/or HLA class I loci [100]. By targeting genomic sequences in constant regions of α and β subunits of TCR or interrupting HLA-A locus, TCR or HLA class I antigens are not capable of expressing and thus T-cells could not recognize allogeneic antigens, resulting in preventing the occurrence of GVHD. The allogeneic T-cells derived from healthy donors are universal T-cells and can be used as universal CAR T-cells for specific antigens of interest (Table 2). 

It is generated by several genome-editing technologies such as zinc finger nuclease (ZFN), transcription activator-like effector nuclease (TALEN), and clustered regulatory interspaced short palindromic receptor (CRISPR)/CRISPR-associated protein 9 (CRISPR/Cas9), which have been used to modify genes and re-engineer cells [101,102,103,104,105]. In addition to the potential of these cells for application in multiple recipients, the universal CAR T-cells can be used to target multiple antigens without the re-editing and production of T cells. 

To enhance the flexibility and controllability of CARs, a split-CAR system named SUPRA CAR was invented. The CAR system is a two-component receptor systemic consisting of a universal receptor with a leucine zipper adapter in T-cells and a separate scFv with a leucine zipper adapter molecule targeting the specific antigen [106]. These versatile systems facilitate the combination of universal CAR T-cells with zipFvs targeting different antigens to overcome antigen loss.

## 16. Conclusions

The CD19 CAR T-cell therapy is a promising therapeutic strategy that yields dramatic improvement in patients with relapse or in preclinical and clinical studies of refractory B-cell hematologic malignancies. However, numerous data in Table 1 demonstrated that cell therapy is more effective in B-ALL than B-NHL. Although accurate meta-analysis data was absent, CD19 negative relapse rate in Table 1 is thought to be relatively lower in B-ALL than B-NHL. Therefore, further meta-analysis data about efficacy and relapse rates of cell therapy in B-cell lineage hematologic malignancies would be needed.

CAR T-cells offer the prospect of improved targeting, with the potential for durable outcomes. Nevertheless, resistance mechanisms such as antigen-positive relapse and antigen loss were identified, and need to be addressed. Limited CAR T-cell persistence or transient B-cell aplasia after cell therapy is associated with antigen-positive relapse. Thus, various treatment approaches for longer antigen expansion and persistence, such as application of CAR-T cells carrying a 4-1BB co-stimulatory domain, administration of AAPCs, central memory T-cells or stem cell-like memory T-cells, and lymphodepletion preconditioning were developed. However, the efficacies of the above treatments were still unclear. Recently, combination therapies with other immunotherapeutic agents associated with T cell co-inhibitory receptors such as PD-1/PD-L1, Tim-3, LAG-3 have been developed. Thus, clinical trials about diverse therapeutic strategies to prevent antigen-positive relapse should be developed.

Meanwhile, the loss of CD19 occurs via a variety of mechanisms, including genetic mutations leading to partial or complete antigen loss, alternative splicing, epitope masking, decreased CD19 antigen density, and lineage switch. Regardless of the antigen-negative resistance mechanism, it is possible that multi-targeted CAR T-cell therapies, such as dual and tandem CAR T-cells, may overcome the resistance and increase survival. Armored CAR T-cells show more effective antitumor activity due to additional cytokine secretion. Moreover, universal CAR T-cells show versatile functions to overcome resistance as donor-derived allogeneic CAR-T cells against multiple antigens. However, clinical data efficacy and side effects about dual, tandem, and universal CAR T-cell therapy in B-cell hematologic malignancies are still limited. Thus, more clinical data is needed to confirm the efficacies of the agents. Subsequently, improved diverse treatment strategies are needed to overcome the resistance and improve clinical outcomes in relapsed and refractory B-cell hematologic malignancies.

## Figures and Tables

**Table 1:** Clinical data of CD19 chimeric antigen receptor (CAR) T-cell therapy in B-cell malignancies.

Study	Patients (*n*)	Co-stimulatory Domain	Lymphodepletion Regimen	Response Rate	Relapsed or not Responded Rate	CD19 (-) Relapse Rate (%)	Reference
**B-ALL**							
CHOP (Maude et al.)	Pediatric and adult B-ALL (30)	4-1BB	Investigator’s choice	CR, 27 of 30 (90%)	8 of 27 (29.6%)	3 of 27 (11.1%)	[10]
ELIANA (Maude et al.)	Pediatric and young Adult B-ALL (75)	4-1BB	Flu-Cy/Cytarabine-etopo	CR, 61 of 75 (81%)	17 of 61 (27.9%)	15 of 61 (24.6%)	[11]
NCI (Lee et al.)	Pediatric and adult B-ALL (21)	CD28	Flu-Cy/FLAG/Ifosfamide-etopo	CR, 14 of 21 (66.7%)	7 of 14 (50%)	2 of 14 (14.3%)	[12]
SCRI (Gardner el al.)	Pediatric and adult B-ALL (7)	4-1BB	Flu-Cy/Cy only	CR, 100%	2 of 7 (28.6%)	2 of 7 (28.6%), lineage-witch	[13]
MSKCC (Park et al.)	Adult B-ALL (53)	CD28	Flu-Cy/Cy only	CR, 44 of 53 (83%)	25 of 44 (57%)	4 of 44 (9%)	[14]
FHCRC (Turtle et al.)	Adult B-ALL (29)	4-1BB	Flu-Cy/Cy only	CR, 27 of 29 (93%)	9 of 27 (33.3%)	2 of 27 (7.4%)	[15]
**B-NHL**							
ZUMA-1 (Neelapu et al.)	DLBCL, PMBCL, tFL (111)	CD28	Flu-Cy	ORR, 82 % (CR, 54%)	52 of 111 (46.8%)	3 of 11 analyzed (27.2%)	[16]
Schuster et al.	DLBCL, FL (28)	4-1BB	Investigator’s choice	ORR, 52 % (CR, 40%)	5 of 28 (17.9%)	1 of 5 analyzed, (20%)	[17]
Jacobson et al.	Aggressive B-NHL (73)	CD28	unknown	ORR, 57 % (CR, 36%)	Unknown	1 of 4 analyzed, (25%)	[18]
Oak et al.	DLBCL, PMBCL, tFL (22)	CD28	unknown	ORR, 86 % (CR, 45.5%)	5 (22.7%)	2 of 4 analyzed, (50%)	[19]

**Table 2:** Therapeutic strategies to overcome CD19 antigen-negative relapse.

CAR Type	Characteristics	References
**Dual targeted CAR T-cell therapy**		
Dual CAR	Dual CARs consist of two separate second-generation CARs targeting two different tumor antigens. The dual CARs can be classified in “bi-cistronic CAR” type expressing two CARs such as CD19 and CD22 simultaneously and “mono-CAR” type expressing the two CARs separately.	[83,84,85,86]
Tandem CAR	A single CAR molecule is incorporated into two different binding molecules with different antigen specificities.	[61,87,88,89,90]
**Armored CAR**		
	CAR T-cells with mechanisms to allow for local delivery of cytokine to enhance antitumor activity of T cells but to reduce activities of immune suppressor cells and mitigate potential toxicity. TRUCKs: T-cells redirected for universal cytokine-mediated killing -CAR T-cells lead to release IL-12 upon engagement with cancer -leads to recruitment of activated macrophages, inflammation, and cancer cell lysis with antigen loss.	[31,91,92,93,94,95,96,97,98,99]
**Universal CAR**		
	In several genome editing techniques (ZFN, TALEN, and CRISPR/Cas9) to modify gene and re-engineer cells, universal CAR could be knocked out TCR and HLA. The optimal universal CAR might be HLA and TCR negative and include non-classical HLA to avoid NK cell lysis. SUPRA CAR: consists of zipCAR linked to B leucine zipper and zipFv linked to A leucine zipper. Through binding between A and B leucine zippers, any extracellular signal linked to the A leucine zip can activate the T-cells.	[100,101,102,103,104,105,106]
